# A new insight on evaluation of the fertility and pregnancy outcome in patients with primary Sjögren syndrome: a propensity score matched study in multi-IVF centers

**DOI:** 10.1186/s12958-024-01228-4

**Published:** 2024-05-20

**Authors:** Ruolin Mao, Lixia Zhu, Rui Long, Juepu Zhou, Xiangfei Wang, Meng Wang, Tiantian Wang, Youzhu Li, Hui Long, Lei Jin

**Affiliations:** 1 Reproductive Medicine Center, Tongji Hospital, Tongji Medical College, Huazhong University of Science and Technology, Wuhan, China; 2 Department of Assisted Reproduction, Shanghai Ninth People´S Hospital, Shanghai Jiao Tong University School of Medicine, Shanghai, 200011 China; 3 Department of Reproductive Medicine, The First Affiliated Hospital of Xiamen University, School of Medicine, Xiamen University, Xiamen, China; 4 Laboratory of Medical Molecular Biology, Si Ming Branch, The First Affiliated Hospital of Xiamen University, School of Medicine, Xiamen University, Xiamen, China

**Keywords:** Primary Sjögren syndrome, Female fertility, Oocyte and embryonic development, Pregnancy outcomes, In vitro fertilization

## Abstract

**Background:**

Primary Sjögren syndrome (pSS) is often related to adverse neonatal outcomes. But it’s currently controversial whether pSS has an adverse effect on female fertility and clinical pregnancy condition. More importantly, it’s unclear regarding the role of pSS in oocyte and embryonic development. There is a lack of comprehensive understanding and evaluation of fertility in pSS patients.

**Objective:**

This study aimed to investigate oocyte and embryonic development, ovarian reserve, and clinical pregnancy outcomes in Primary Sjögren syndrome (pSS) patients during in vitro fertilization (IVF) treatment from multi-IVF centers.

**Methods:**

We performed a muti-central retrospective cohort study overall evaluating the baseline characteristics, ovarian reserve, IVF laboratory outcomes, and clinical pregnancy outcomes between the pSS patients and control patients who were matched by Propensity Score Matching.

**Results:**

Following PSM matching, baseline characteristics generally coincided between the two groups. Ovarian reserve including anti-müllerian hormone (AMH) and antral follicle counting (AFC) were significantly lower in the pSS group vs comparison (0.8 vs. 2.9 ng/mL, *P* < 0.001; 6.0 vs. 10.0, *P* < 0.001, respectively). The pSS group performed significant reductions in numbers of large follicles, oocytes retrieved and MII oocytes. Additionally, pSS patients exhibited obviously deteriorate rates of oocyte maturation, 2PN cleavage, D3 good-quality embryo, and blastocyst formation compared to comparison. As for clinical pregnancy, notable decrease was found in implantation rate (37.9% vs. 54.9%, *P* = 0.022). The cumulative live birth rate (CLBR) following every embryo-transfer procedure was distinctly lower in the pSS group, and the conservative and optimal CLBRs following every complete cycle procedure were also significantly reduced in the pSS group. Lastly, the gestational weeks of the newborns in pSS group were distinctly early vs comparison.

**Conclusion:**

Patients with pSS exhibit worse conditions in terms of female fertility and clinical pregnancy, notably accompanied with deteriorate oocyte and embryo development. Individualized fertility evaluation and early fertility guidance are essential for these special patients.

## Introduction

It is common knowledge that immunological factors play a significant role in female infertility, and in recent years, it has drawn more attention [1]. Reproductive failure and autoimmune illnesses are strongly linked, according to a large body of research [2]. Primary Sjögren syndrome (pSS) is a systemic autoimmune disease without any other autoimmune diseases characterized by the involvement of exocrine glands mainly salivary and lacrimal glands, resulting in a range of clinical manifestations from sicca symptoms to multi-system damage, such as pulmonary interstitial fibrosis, cirrhosis, and lymphoma [3]. This disease predominantly affects middle-aged and subsequent women, but can also be observed in children, men and the elderly [4]. In general, women with pSS may experience a more complex pregnancy process, leading to a higher probability of gynecological complications or adverse neonatal outcomes [5–7]. Prior and derivative works indicated that elevated anti-Sjögren Syndrome A (anti-SS-A) antibodies in SS patients was associated with risk of congenital heart block [8]. In addition, a lower birthweight of offspring and a higher caesarean section rate in primary SS women were more commonly observed [9].

However, the relationship between pSS and women's fertility remains controversial. More importantly, the role of Sjögren syndrome in oocyte and embryonic development remains to be explored. One reason is that, because of physiological restrictions, direct observations of oocyte and embryonic development are unavailable, leading to a paucity of data on the oocyte and embryonic development in women with pSS. However, the development of in vitro fertilization and embryo transfer (IVF-ET) technology has made it feasible to visualize oocyte and embryos development in vitro. Furthermore, some previous investigations have indicated a decline in ovarian reserve among women who have been diagnosed with SS [10] and proposed that an increased risk of spontaneous abortion could be attributed to pSS [11]. However, some research revealed that the pregnancy outcomes of primary SS patients and controls were comparable [9, 12]. Data on women fertility and embryonic development of patients with Sjögren syndrome are indeed scarce and uncertain. To clarify, more research on this field is therefore required.

The present study systematically assessed the oocyte and embryonic development, ovarian reserve, clinical outcomes and neonatal outcomes between pSS patients and the comparisons. With the first-hand evidence of oocyte and embryonic development, this study could help us fully understand the fertility of pSS patients from multiple angles, and provide support for embryologists and clinicians in dealing with pSS patients counseling and providing fertility instruction more thoughtfully and rationally.

## Material and methods

### Study design and population

This was a muti-center retrospective cohort study. Women undergoing in vitro fertilization /intracytoplasmic sperm injection (IVF/ICSI) cycles from January 2014 to August 2023 in the three university affiliated hospitals were reviewed. Forty-seven women with a history of rheumatologically confirmed primary Sjogren syndrome were included in the pSS group. All included pSS patients were clinically diagnosed as pSS in the department of rheumatology before they were treated with ART. The classification criteria for pSS were based on the 2016 American College of Rheumatology/European League Against Rheumatism (ACR/EULAR) classification criteria [13]. After comprehensive evaluation by rheumatologists and reproductive clinicians, IVF could be performed if pSS patients had the lowest disease activity, normal immune indicators or lowest antibody titers, and pSS patients are not taking drugs or taking drugs with minimal effect and maintaining more than three months.

**Fig. 1:**
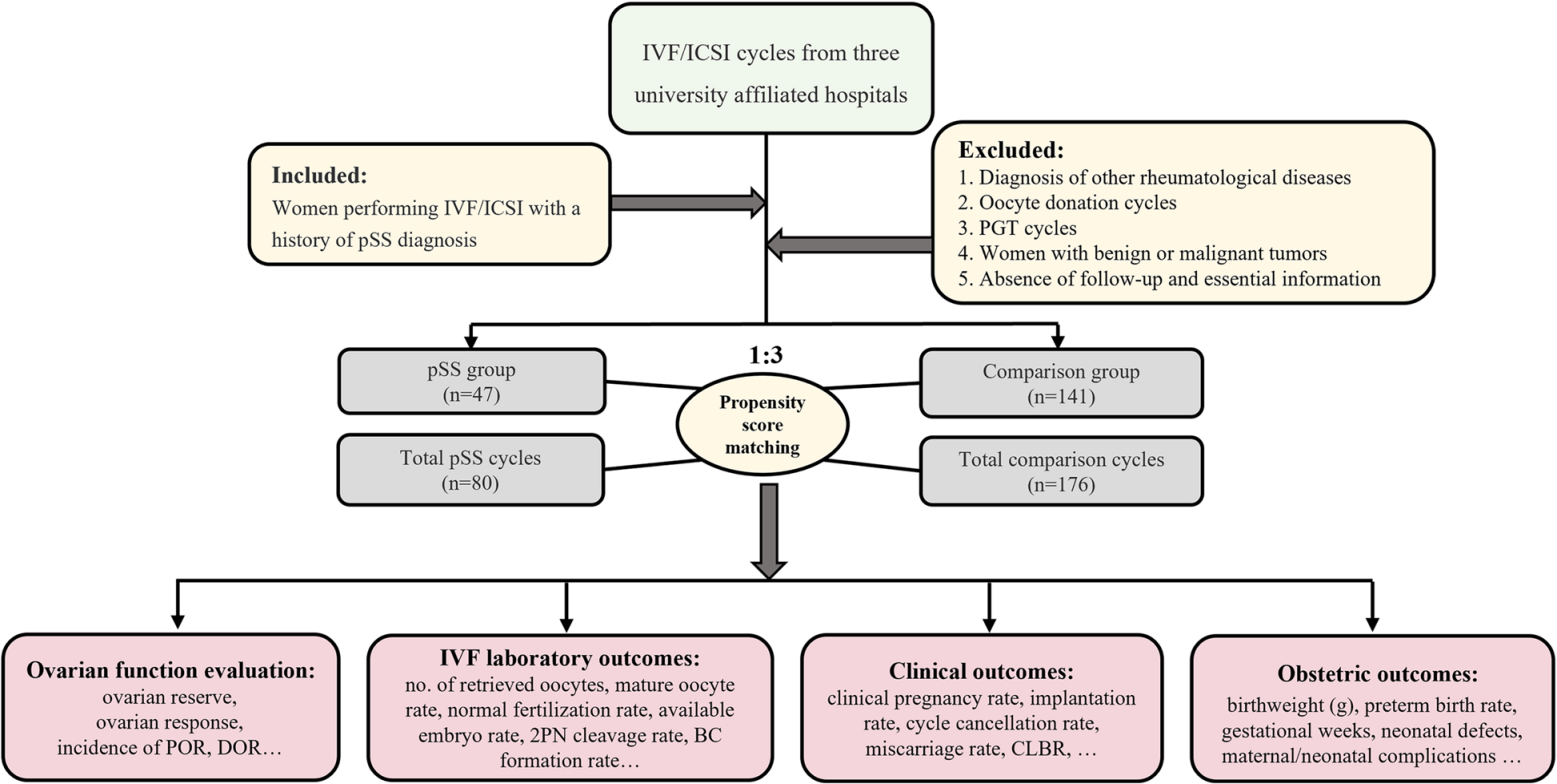
Flow chart of the present study. IVF: in vitro fertilization, ICSI: intracytoplasmic sperm injection; pSS: Primary Sjögren syndrome; PGT: preimplantation genetic testing; POR: poor ovarian response; DOR: diminished ovarian reserve; 2PN, 2 pronucleus; BC: blastocyst; CLBR: cumulative live birth rate

The criteria for exclusion were as follows: (1) diagnosis of other rheumatological diseases; (2) oocyte donation cycles; (3) preimplantation genetic testing (PGT) cycles; (4) women with benign or malignant tumors; (5) absence of follow-up and essential information. A propensity score matching (PSM) of 1:3 ratio was carried out to balance the distribution of sample and clinical characteristics, taking into account the complexity of the etiology in pSS patients undergoing assisted reproductive technique (ART) and to remove the potential confounders in the pSS patients and comparisons. Several parameters such as age of infertile patients, BMI, type of infertility, duration of infertility, and cause of infertility were included in the PSM matching. In particular, the comparison was also matched when pSS patients had conditions that could have an impact on their ovarian reserve, such as endometriosis, polycystic ovarian syndrome (PCOS), and ovarian surgery. All comparison patients denied a history of pSS disease and did not find any conscious symptoms of pSS such as xerostomia and xerophthalmia after follow-up. The routine immunization suite of the comparison patients did not reveal any abnormality related to pSS. Details about the patients' enrollment and comparison were shown in Fig. 1.

### Ovarian stimulation protocol, oocyte retrieval, and embryo transfer

Protocols for ovarian stimulation were processed as previously described [14]. To put it briefly, protocols including the gonadotropin-releasing hormone (GnRH) agonist and antagonist protocols, as well as other protocols like the mild stimulation and luteal phase stimulation protocols were used. Recombinant follicle-stimulating hormone (FSH) dosage and duration were modified based on each patient's unique ovarian response. Transvaginal ultrasonography was used to track the diameter of the follicle. Recombinant human chorionic gonadotropin (HCG) was injected intramuscularly as a trigger when the diameter of two or three dominant follicles surpassed 18 mm. Oocytes were then extracted 36–38 h following HCG injection through guided transvaginal ultrasonography. Embryos may be transferred on day 3 as appropriate after oocyte retrieval. The remaining available embryos could either be frozen on day 3 or further cultured to day 5 or day 6 for cryopreservation. Cryopreserved embryos were transferred after priming the uterus with estrogen.

### Data collection

The main outcomes evaluated in the current study were demographic characteristics, ovarian reserve, oocyte/embryo developmental information in vitro, clinical pregnancy outcomes, and obstetric outcomes as well as maternal complications. For demographic characteristics, the age at cycle start, body mass index (BMI), infertility type, infertility duration, and causes of infertility were gathered. Antral follicle count (AFC), anti-müllerian hormone (AMH) level, and basal serum FSH level were indicators of ovarian reserve. IVF/ICSI cycle information included the amount of gonadotropin used, total days of ovarian stimulation, estradiol (E2) level, number of large follicles on HCG trigger day, the incidence of DOR/POR, laboratory outcomes (number of oocytes retrieved, matured, fertilized, available embryos, blastocysts, good-quality embryo, embryos transferred) as well as clinical outcomes (cycle cancellation rate, the number of implantations, miscarriages, pregnancies, and cumulative pregnancies as well as cumulative live births). Lastly, Obstetric outcomes (the number of live birth, birthweight, gestational weeks, preterm birth, cesarean section rate) and maternal/neonatal complications (placental abnormalities, hypertensive disorder, gestational diabetes, neonatal defects, pathologic jaundice and neonatal pneumonia) were also included.

### Criteria of assessment

The Bologna criteria [15] were used to classify women with poor ovarian response (POR) if they had at least two of the following characteristics: an abnormal ovarian reserve test (AFC < 5–7 or AMH < 0.5–1.1 ng/ml), advanced maternal age (≥ 40 years) or any other risk factor for POR, and POR in a previous cycle (≤ 3 oocytes with a conventional ovarian stimulation protocol). Women diagnosed with DOR should exist at least two of the following features: advanced maternal age (≥ 40 years); basal FSH ≥ 12mIU/mL; AFCs ≤ 5–7; AMH ≤ 1.1 ng/ml. The normal fertilization rate was defined as the zygotes of two pronuclei (2PN) numbers divided by the number of yield oocytes; the 2PN cleavage rate was the number of 2PN cleaved embryos divided by the number of 2PN zygotes; the available embryo rate referred to the ratio of the number of embryos available for transfer, cryopreservation, and extended culture to day 3 divided by the number of normally-fertilized and cleaved embryos plus the late-cleaved embryos; the blastocyst formation rate was defined as the number of blastocysts divided by the number of day 3 embryos for extended culture; the good-quality blastocyst formation rate referred to ratio of the blastocysts available for cryopreservation divided by the number of day 3 embryos for extended culture. Cycle cancellation was defined as one cycle without available oocytes after the ovarian stimulation. The implantation rate was the ratio of the number of gestational sacs divided by the number of embryos transferred. Biochemical pregnancy was defined as a positive result of HCG measurement in the 12 to 14 days after embryo transfer. Clinical pregnancy was confirmed if an intrauterine fetal heartbeat could be observed by transvaginal ultrasound. Live birth was defined as the birth of at least one live child after 28 weeks of gestation. The loss of fetal heart activity within 12 weeks after confirming clinical pregnancy was regarded as early miscarriage. Diseases during the gestation period such as gestational diabetes, hypertensive disorders complicating pregnancy, and placental abnormalities were included in the maternal complications. The obstetric outcomes comprised birthweight (g), gestational week, and cesarean section (only singletons). Pre-term birth was defined as a gestational age less than 37 weeks, and low birthweight was referred to birthweight below 2500 g.

Deliveries of multiple pregnancies were only counted as one live birth. For the first-cycle cumulative live birth rate (CLBR), live birth rates (LBR) were calculated following every embryo transfer procedure during the first complete cycle. Two different types of CLBRs were calculated up to final cycles [16]. The optimal CLBR assumed that women who discontinued ART treatment would have had the same chance of having a live birth with continued ART as those who did continue, compared to the conservative CLBR, which was calculated based on the assumption that women who discontinued ART treatment would not have achieved a live birth if they had continued [17]. Women were deemed to terminate ART treatment if they failed to have a treatment-dependent live birth and did not return for any more ART cycles until August 1, 2023.

### Statistical analysis

Data were analyzed and presented using Statistical Package for Social Sciences software (SPSS, version 22.0, IBM, the United States) and R (version 4.1.3). Kruskal–Wallis nonparametric method was performed when dealing with continuous data. The results were reported as median (interquartile range IQR), or with a student t-test if variables were normally distributed. The frequency (percentage, %) and number of cases for categorical data were displayed, and group differences were evaluated using the Chi-Square test. The following baseline characteristics were matched by propensity score matching: age (years), body mass index (BMI, kg/m^2^), infertility type (primary or secondary), infertility period (years), infertility causes (male, female, or combined), previous existing ovarian related diseases such as endometriosis, PCOS and ovarian surgery. With a caliper value of 0.1, the match ratio was 1:3 and matching algorithm adopted the nearest neighbor random matching without replacement. The conservative CLBR estimate was calculated as the number of live births up to and including a specific treatment cycle, divided by the number of women who started their first ART cycle during the study period. After all treatment cycles were included in the analysis, the Kaplan–Meier approach was used to determine the best estimate of CLBR. Log-rank test and Kaplan–Meier curves with live birth considered as an event were used to illustrate differences between groups [18]. Two-sided Wald P-values were used, with *P* < 0.05 denoting significance.

## Results

### Baseline characteristics

A total of 47 women diagnosed with pSS, involving 80 IVF/ICSI cycles, were identified and enrolled in the pSS group. All 47 patients (100%) were assessed to be in clinical remission. In addition, of the 47 patients included in the pSS group, all patients (100%) were treated with 200 to 400 mg/day of hydroxychloroquine, and 41 patients (87.2%) were treated with prednisone at a maintenance dose of 5 to 10 mg/d during IVF procedures. Two patients (4.3%) had previously undergone methotrexate therapy, but both had stopped for more than one year by the time they started IVF treatment and neither of them had a successful live birth. Eight patients (17.0%) had prior nephritis and most of the clinical manifestations were mild, mainly as asymptomatic hematuria and proteinuria. Of the 15 individuals who ultimately had live births, 10 patients (66.7%) showed an SSA/SSB antibody titer of 1:100 or more during pregnancy, and 5 patients (33.3%) showed an SSA antibody titer of 1:100 or more while being negative for SSB antibodies.

**Fig. 2:**
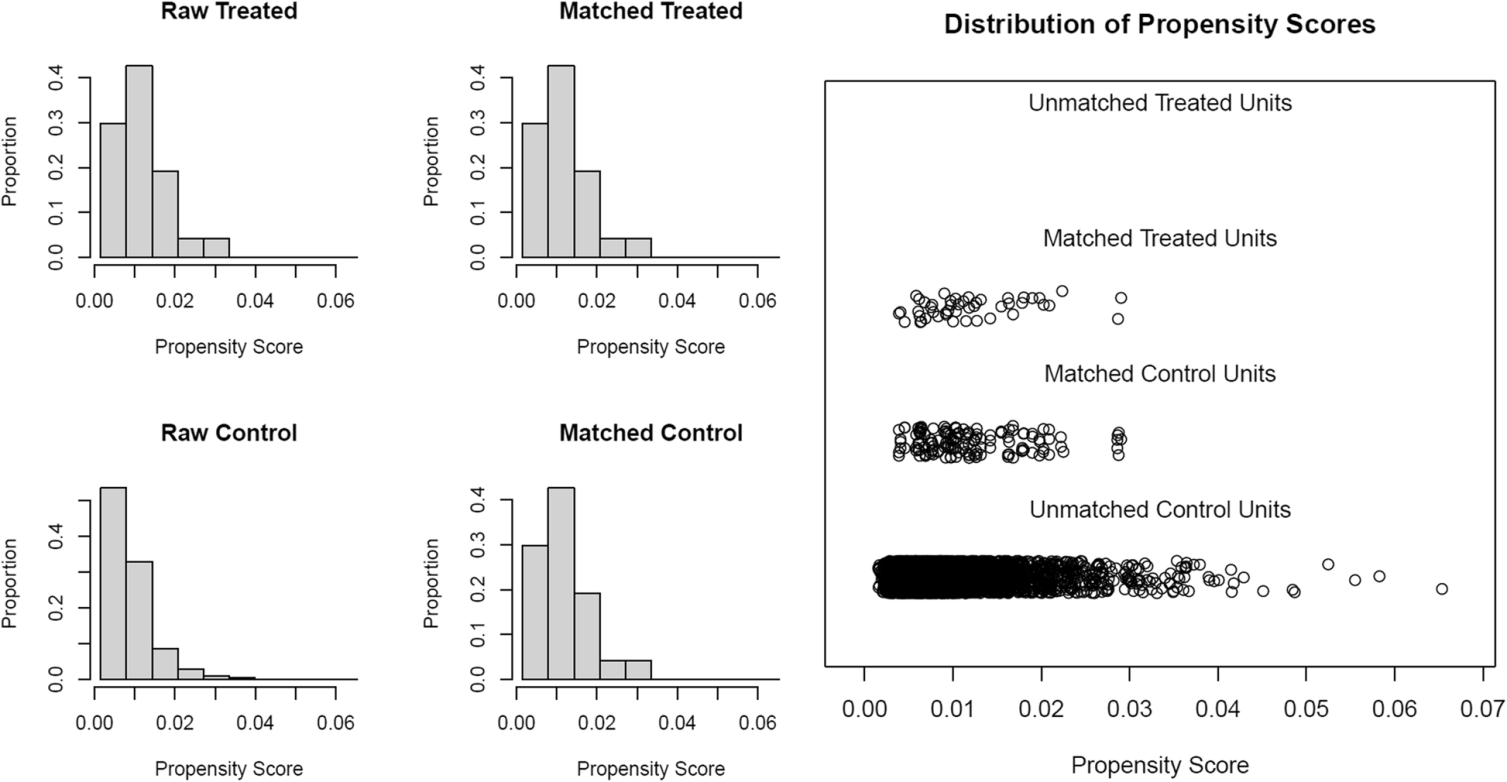
The pre- and post-matching distributions of baseline characteristics and the PSM propensity scores. **A** the baseline characteristic proportion distributions in two groups, before and after PSM matching. **B** Propensity score distributions in two groups before and after PSM matching

Women matched by PSM according to a 1:3 match ratio were included in the comparison group, resulting in 141 patients with 176 IVF/ICSI cycles (Fig. 1). The median age of women at the start of ART was 33.0 years, and the number of primary and secondary types was similar. Other baseline characteristics, including BMI, type of infertility, duration, and cause of infertility, were similar between the two groups (Table 1). The distributions of baseline characteristics and propensity scores were presented through visualized graphics. The proportions of baseline characteristics after matching were almost identical, and the distribution of propensity scores almost coincided between the two groups after matching, confirming the validity of PSM matching (Fig. 2).

**Table 1:** Demographic characteristics of the pSS and comparison groups

Characteristics	pSS patients	Comparison	*P*-value
Number of patients, n	47	141	/
Number of ART cycles, n	80	176	/
Female age at cycle start (y)	33.0(31.0–36.0)	33.0 (31.0–36.0)	0.754
BMI (kg/m^2^)	20.9(18.7–22.4)	21.0 (19.5–23.1)	0.783
Infertility duration (y)	3(1.0–5.0)	3 (2.0–5.0)	0.450
Infertility type, n (%)			1.000
Primary	26(55.3%)	78(55.3%)	
Secondary	21(44.7%)	63(44.7%)	
Infertility cause, n (%)			0.982
Female factors only	23 (48.9%)	71 (50.4%)	
Male factors only	0	2 (1.4%)	
Combined male and female factors	20 (42.6%)	55 (39.0%)	
Unexplained	4 (8.5%)	13 (9.2%)	

Values are median (IQR) for continuous variables and n (%) for categorical variables

*pSS* primary Sjögren syndrome, *ART* Aassisted reproductive technology, *BMI* Body mass index

### Ovarian reserve and response

According to the results, the pSS group had a significantly lower basal AMH level (0.8 vs. 2.9 ng/mL, *P* < 0.001), than the comparison (Table 2). And the AFC was distinctly lower (6.0 vs. 10.0, *P* < 0.001) in the pSS group compared to the comparison. The incidence of DOR in the pSS group was slightly higher than that in the comparison group (36.2% vs 22.0%, *P* = 0.053). As for the ovarian response, patients in the pSS group exhibited significantly lower estrogen level even undergoing similar days and total dose of gonadotropins during controlled ovarian hyperstimulation (1180.0 vs. 1919.5 pg/mL, *P* = 0.009). Additionally, the large follicles of pSS patients on hCG day were obviously lesser than comparison patients (3.0 vs. 9.0, *P* < 0.001). Based on the Bologna criteria, significantly more women with a history of pSS were diagnosed as POR than those without pSS (40.4% vs. 22.7%, *P* = 0.018). The rest of the results were generally consistent between the two groups (Table 2).

**Table 2:** Ovarian reserve and response to stimulation in the pSS and comparison groups

Reproductive results	pSS patients	Comparison	*P*-value
Number of patients, n	47	141	/
Ovarian reserve			
Day3 FSH	7.6 (6.0–10.4)	7.3 (6.2–9.0)	0.351
Day3 AFC	6.0 (4.0–11.0)	10.0 (6.0–16.0)	**< 0.001**
AMH (ng/mL)	0.8 (0.3–2.4)	2.9 (1.5–4.8)	**< 0.001**
Incidence of DOR	36.2% (17/47)	22.0% (31/141)	0.053
Ovarian response			
Total dose of gonadotropins (IU)	2025.0 (1500.0–2850.0)	2100.0 (1575.0–2700.0)	0.193
Days of gonadotropins use (d)	9.0(8.0–10.0)	9.0(8.0–10.0)	0.986
E2 on hCG trigger day (pg/mL)	1180.0(531.0–3515.5)	1919.5 (1209.3–3141.8)	**0.009**
No. of large follicles on hCG day	3 (1–7)	9 (5–13)	**< 0.001**
Incidence of POR	40.4% (19/47)	22.7% (32/141)	**0.018**

Bold fonts were statistically significant

*pSS* primary Sjögren syndrome, *FSH* Follicle-stimulating hormone, *AFC* Antral follicle count, *AMH* Antimüllerian hormone, *DOR* Diminished ovarian reserve, *E2* Estradiol, *hCG* human chorionic gonadotropin, *POR* Poor ovarian response

### Oocyte and embryo viability assessment

Regarding the IVF laboratory outcomes, number of oocytes retrieved (5.0 vs. 9.0, *P* < 0.001) and numbers of MII oocytes (3.0 vs. 8.0, *P* < 0.001) in the pSS group were distinctly fewer than the comparison. Hence, there were certain differences in the maturation rate between the pSS group and the comparison (81.2% vs 85.8%, *P* = 0.005). In addition, the 2PN cleavage rate were markedly different between the two groups (98.2% vs. 99.5%, *P* = 0.022), while the normal fertilization rate and available embryo rate was comparable between the two groups. It is worth noting that following the embryo extended culture, significant differences were observed in the D3 good-quality embryo rate (39.3% vs. 50.1%, *P* < 0.001), blastocyst formation rate (52.9% vs. 72.3%, *P* < 0.001) and good-quality blastocyst formation rate (33.3% vs. 48.1%, *P* < 0.001) between pSS group and the comparison (Table 3).

**Table 3:** IVF/ICSI laboratory outcomes and clinical outcomes of pSS and comparison groups

IVF/ICSI outcomes	pSS patients	Comparison	*P*-value
Number of ART cycles, n	80	176	/
Laboratory outcomes			
No. of oocytes retrieved per patient	5 (2–11)	9 (6–14)	** < 0.001**
No. of MII oocytes per patient	3 (1–9)	8 (5–12)	** < 0.001**
Maturation rate	81.2% (519/639)	85.8% (1601/1865)	**0.005**
Normal fertilization rate	61.7% (394/639)	64.3% (1199/1865)	0.233
2PN Cleavage rate	98.2% (387/394)	99.5% (1193/1199)	**0.022**
Available embryo rate	82.9% (374/451)	83.7% (1186/1417)	0.701
D3 good-quality embryo rate	39.3% (155/394)	50.1% (601/1199)	** < 0.001**
Blastocyst formation rate	52.9% (135/255)	72.3% (709/980)	** < 0.001**
Good-quality blastocyst formation rate	33.3% (85/255)	48.1% (471/980)	** < 0.001**
Clinical outcomes			
Cycle cancellation rate	47.5% (38/80)	9.7% (17/176)	** < 0.001**
No. of ET cycles	68	258	/
No. of embryos transferred	105	297	/
Average no. of embryos transferred	1.5 ± 0.5	1.2 ± 0.4	**0.001**
Implantation rate	25.7% (27/105)	42.8% (127/297)	**0.002**
Biochemical pregnancy rate	50.0% (34/68)	53.9% (139/258)	0.569
Clinical pregnancy rate	39.7% (27/68)	49.2% (127/258)	0.162
Early miscarriage rate	18.5% (5/27)	18.1% (23/127)	0.960
LBR per transfer cycle	22.1% (15/68)	36.8% (95/258)	**0.022**
LBR per complete cycle	35.7% (15/42)	59.7% (95/159)	**0.005**

Bold fonts were statistically significant

*pSS* primary Sjögren syndrome, *IVF/ICSI* In vitro fertilization/intracytoplasmic sperm injection, *ART* Assisted reproductive technology, *MII* Metaphase II, *2PN* 2 pronucleus, *ET* Embryo transfer, *LBR* Live birth rate

### Clinical outcomes

Due to the deteriorated embryo development, 21 patients in the pSS group had to cancel a total of 38 cycles due to lack of available embryos, and the cycle cancellation rate was 47.5%, compared with 13 patients in the comparison group with a total of 17 cycles, manifesting only 9.7% of the cycle cancellation rate (*P* < 0.001). For all embryo transfer cycles, the average numbers of transferred embryos were 1.5 and 1.2 in the pSS and comparison groups, respectively (*P* = 0.001). And our results showed a notably lower implantation rate in the pSS group (37.9% vs. 54.9%, *P* = 0.022), whereas the rates of biochemical pregnancy, clinical pregnancy and early miscarriage were generally consistent between the two groups.

**Fig. 3:**
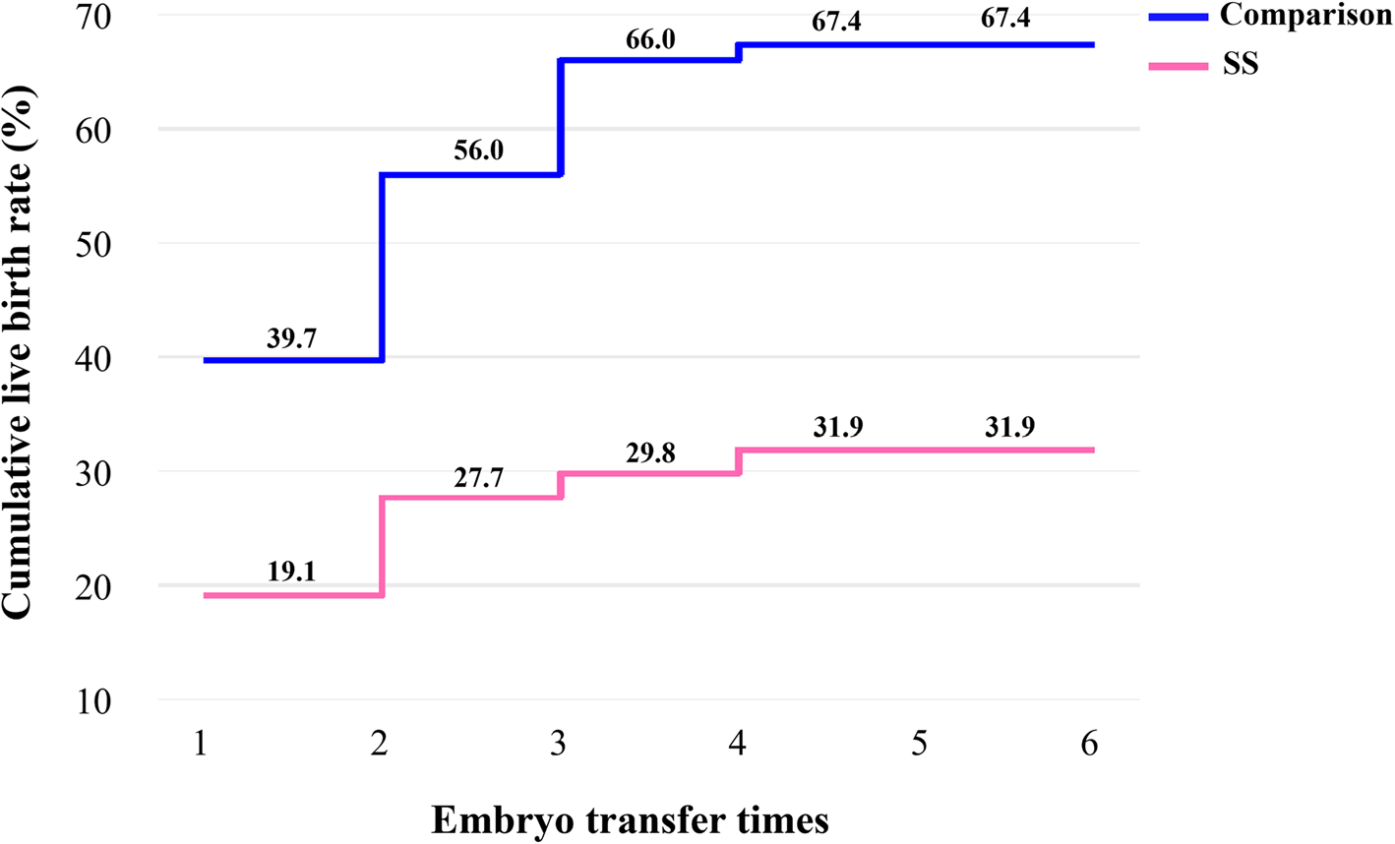
The CLBR following every embryo-transfer procedure. For each complete cycle, the LBR following every embryo-transfer procedure rose from 19.1% to 31.9% in the pSS group (red line), and from 39.7% to 67.4% in the comparison group (blue line). Significant differences were found between the two groups (*P* < 0.001)

**Fig. 4:**
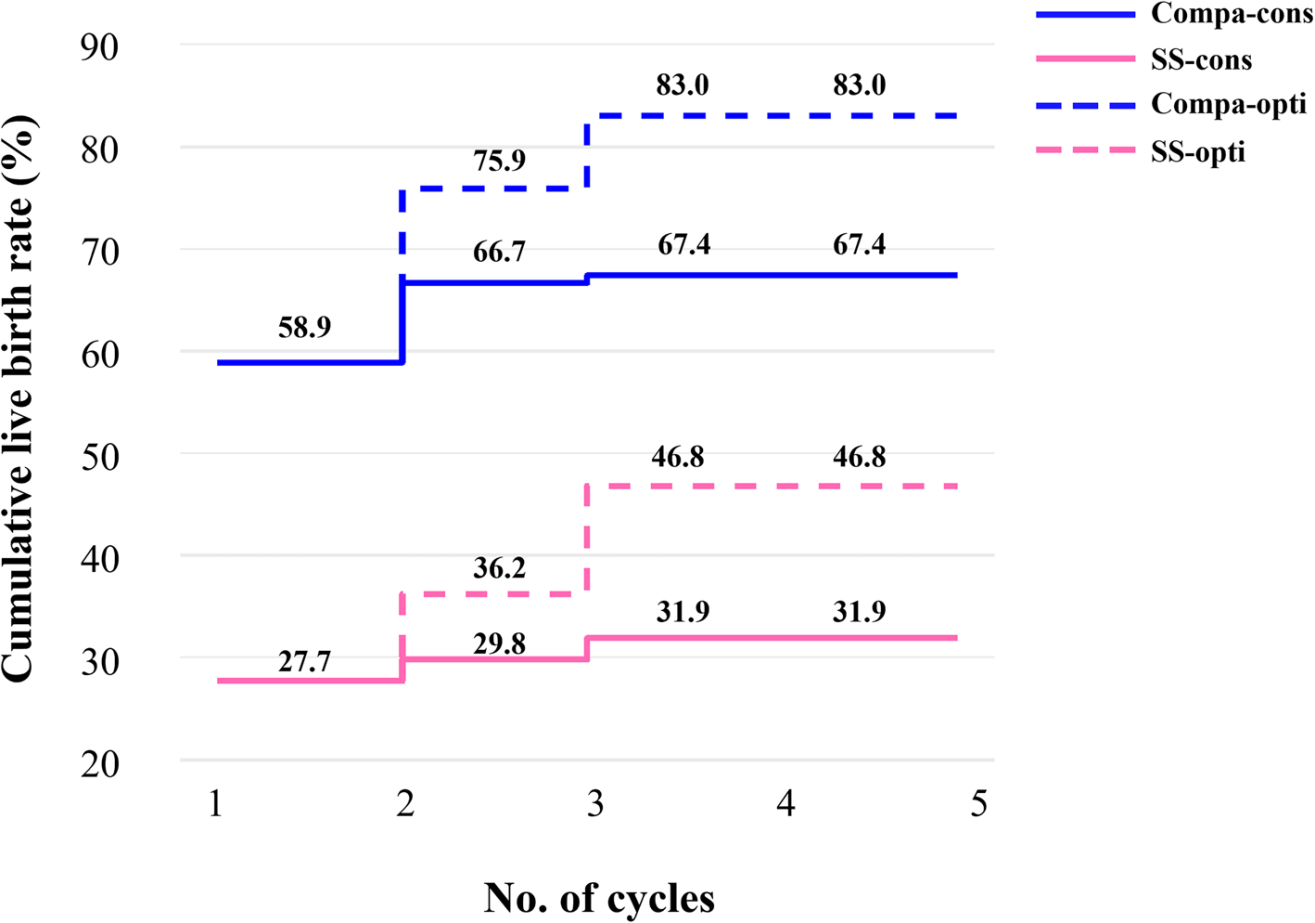
The conservative and optimal CLBRs for up to five complete cycles in two groups. The CLBR was 27.7% for the first complete cycle of the pSS group, rising to 31.9% (conservative) and 46.8% (optimal) for the fifth cycle. In the comparison group, the CLBR increased from 58.9% for the first cycle to 67.4% (conservative) and 83.0% (optimal) for the fifth cycle. The difference of optimal CLBRs between the two groups was significant (*P* < 0.001). CLBRs did not increase from the fourth cycle in the pSS group

For one complete cycle, which involves the outcomes from all fresh and following frozen/thawed embryo transfers after one ovarian stimulation, the LBR of the pSS group were distinctly lower than those of the comparison group (35.7% vs. 59.7%, *P* = 0.005) (Table 3). For all transfer cycles, which included the fresh and frozen/thawed embryo transfers of all the patients, the LBR per transfer cycle of the pSS group were also significantly lower than comparison (22.1% vs. 36.8%, *P* = 0.022). For per complete cycle, the CLBR following every embryo-transfer procedure increased from 19.1% to 31.9% in the pSS group, and from 39.7% to 67.4% in the comparison group (*P* < 0.001), as shown in Fig. 3. The conservative and optimal CLBRs for up to fifth complete cycles were presented in Fig. 4. In general, the CLBR of the first complete cycle in the pSS group was 27.7%, later rising to 31.9% (conservative) and 46.8% (optimal) for the fifth complete cycle, while in the comparison group, the CLBR rose from 58.9% for the first cycle to 67.4% (conservative) and 83.0% (optimal) for the fifth cycle. The difference in conservative and optimal CLBRs between the two groups was significant (*P* < 0.001, both). CLBRs did not increase from the fourth complete cycle in the pSS group (Fig. 4).

### Obstetric and maternal outcomes

Of the 47 pSS patients with a total of 15 live birth events, including 11 singletons pregnancies and 4 twin pregnancies, whereas in the 141 comparison individuals, a total of 95 live birth events included 93 singleton pregnancies and 2 twin pregnancies. The obstetric characteristics including birthweight, low birthweight, gestational age, pre-term birth, as well as cesarean section (only singleton pregnancy was counted), are detailed in Table 4. There were no significant differences in all the obstetric characteristics except gestational weeks (37.3 vs 38.5 weeks, *P* = 0.009) between pSS group and comparison. The maternal and neonatal complications of live-born were recorded and are presented in Table 4. One placental abnormality and one neonatal defect were observed in the pSS group, whereas two placental abnormalities and one case in both of gestational hypertension and gestational diabetes were observed in the control group. It is worth mentioning that a newborn in the pSS group performed one degree of conduction block and was classified among neonatal defects and was followed up to learn that the SSA antibody titer was as high as 1:1000 during maternal pregnancy. One case in both of pathologic jaundice and pneumonia were observed in the control group, whereas neither were observed in the pSS group.

**Table 4:** Obstetric outcomes, maternal and neonatal complications between the pSS and comparison groups

Characteristics	pSS patients	Comparison	*P*-value
Number of patients, n	47	141	/
Live birth	15	95	**/**
Singletons (n)	11	93	**/**
Twins (n)	4	2	/
Obstetric outcomes			
Birthweight (g)^a^	3007.3 ± 502.9	3196.6 ± 419.8	0.169
Low birthweight (< 2500 g)^a^	18.2% (2/11)	3.2% (3/93)	0.086
Gestational weeks^a^	37.3 ± 1.8	38.5 ± 1.3	**0.009**
Pre-term birth (< 37 weeks)^a^	27.3% (3/11)	9.7% (9/93)	0.114
Cesarean section rate^a^	72.7% (8/11)	84.9% (79/93)	0.382
Maternal complications, n			
Placental abnormalities	1	2	**/**
Hypertensive disorder	0	1	**/**
Gestational diabetes	0	1	**/**
Neonatal complications			
Neonatal defects	1	0	**/**
Pathologic jaundice	0	1	**/**
Neonatal pneumonia	0	1	/

Bold fonts were statistically significant

*pSS* primary Sjögren syndrome

^a^Only singleton pregnancy outcomes were counted

## Discussion

As was previously indicated, advancements in ART alongside heightened awareness have led to a rising trend of infertile patients with pSS seeking ART interventions. However, concerns have escalated regarding the negative effects of pSS antibody on the pregnancy process and the newborn [3]. In addition, few studies have explored the prevalence of pSS in infertile women and the exact impact of pSS on female fertility and its downstream effects. There are also no studies that examine whether women with pSS irreversibly impaire oocyte and embryonic development potential. In the current study, we comprehensively assessed the effect of pSS on oocyte and embryo competence, ovarian reserve, clinical and obstetric outcomes. The results of present study showed that in comparison to women with common infertility, women with diagnosed pSS or a history of pSS had compromised oocyte and embryo viability and lower ovarian reserve, thus resulting in poorer IVF clinical outcomes. We provided a new perspective on fertility assessment in pSS patients in terms of evident observation of oocyte and embryo development.

For one thing, the ovarian function was observed a significantly diminished in pSS patients compared to comparison. Patients with pSS not only exhibited lower AMH levels and AFC counts, but also had a much higher incidence of DOR and POR than comparison. This was in line with the results of a cohort study of a Turkey population, which found the ovarian reserve may be reduced in pSS patients [10]. And some prior studies reported that patients with pSS experienced early menopause or menstrual abnormality more frequently than the general population, which can also be a sign of declined ovarian function [19, 20]. In fact, prior investigations have reported the impact of autoimmune disorders, notably systemic lupus erythematosus (SLE), on female reproductive health, including ovarian reserve impairment and heightened susceptibility to premature ovarian failure [21, 22]. However, in contrast to the extensive scrutiny afforded to SLE in the context of female fertility, the studies concerning pSS remains notably limited. Therefore, this study could provide a valuable supplementary, especially as more and more pSS patients are choosing for ART.

Secondly, as with most autoimmune diseases, environmental and hormonal are implicated in the pathogenesis of pSS. Cumulative evidence suggested low estrogen levels, and localized dihydrotestosterone deficiency appeared to be more prone to pSS [25, 26]. Increasing estrogen exposure could be negatively related with risk of pSS [27]. Our findings also showed a significantly lower E2 level in pSS group even after controlled ovarian hyperstimulation. Of concern is that studies revealed that pSS could be associated with dysfunction of hypothalamic–pituitary–adrenal (HPA) axis and thyroid axes. Distinctly lower basal adrenocorticotropin (ACTH) and cortisol levels were found in patients with pSS [28]. Not only that, but serum prolactin levels may also be elevated in patients with pSS, which are widely considered to be associated with female infertility [29, 30]. Hence, in instances where patients diagnosed with pSS present with the above clinical attributes, it is essential for them to undergo comprehensive assessment by interdisciplinary teams comprising rheumatologists and reproductive specialists. This collaborative approach will facilitate the formulation of tailored treatment strategies aimed at optimizing early fertility outcomes.

In terms of oocyte and embryonic development in patients with pSS, the present study provided valuable insights that have not yet been focused on in previous studies. It is worth mentioning that our results also showed that pSS patients exhibited worse oocyte and embryonic development in terms of oocytes retrieval, maturation, embryo cleavage and blastocyst formation. The potential mechanisms for this could be multifaceted and complex. Primary Sjogren's syndrome is a chronic autoimmune disease with abnormal immunity activation. Over-activated immune cells and a variety of auto-antibodies may play an important role in the process [23, 24], but research is needed to further elucidate. For patients with pSS undergoing ART, the compromised development of oocytes and embryos typically translates to suboptimal treatment outcomes, but so far there has not been widespread concern about the outcome of ART in such patients, and thus there is still no consensus on subsequent interventions strategies. Nonetheless, we still advocate early involvement of rheumatologists and reproductive specialists in the evaluation and management of infertility in patients diagnosed with pSS, taking into account the patient's ovarian reserve function and the development of oocyte and embryos, and integrating a combination of pSS treatment and ART to closely monitor the development of oocytes and embryos in order to achieve the best therapeutic outcome. It's worth noting that our results showed that even pSS was in a remission period, patients still had impaired oocyte and embryo development and poor clinical outcomes. Therefore, any patient with pSS who intended to undergo ART should implement so as soon as possible after evaluation of the disease and try not to let age be a second barrier to fertility.

Additionally, our previous work found that SLE patients also exhibit oocyte and embryonic developmental abnormalities [31], which may imply some commonalities between autoimmune diseases.Delving deeper into the potential mechanisms may enrich our understanding of their complex relationship.In relation to the clinical pregnancy, our study provides a more comprehensive assessment of clinical outcomes, and our results show that even when more embryos are transferred on average, the rates of implantation, LBR per complete cycle, and LBR per transfer are distinctly decreased in pSS patients than in comparison patients. Actually, similar to our findings, a case–control study by Priori et al. reported that the average number of pregnancies in pSS patients were quite lower than normal patients [32]. However, it should be noted that some previous works differ from our results. As early as 1994, a study by Fotini et al. found that the viable infant rate between pSS patients and control were comparable [33]. This conclusion was in line in another study in Denmark, which reported that pregnancy and live births between pSS patients and controls were generally similar [34]. But what has to be mentioned is that above previous studies assessed the pregnancy outcomes of pSS patients included pregnancies before and after diagnosis, representing a possible bias [11, 12, 32–34], whereas our studies only included patients after diagnosis of pSS. It's reasonable to speculate that pSS may exert a relatively small effect on patient with pre-diagnostic pregnancy, which have been confirmed in some subgroup studies. Priori et al. reported that the average pregnancies per woman with the diagnosis of pSS was only 1.27, which was less than the women prior to the diagnosis of pSS, which was two. Moreover, several studies included patients who were menopausal women, and related information may be obtained through questionnaires or oral surveys tracing childbearing history at a young age [11, 33, 34], which has a great potential for bias. In addition, some retrospective studies have simply reported pregnancy rates and live births in patients with pSS without control group [35]. Therefore, conclusions in many previous studies were doubtful due to various bias, while our study offered relatively objective evidence by enrolling clinical diagnosed pSS patients from different hospitals, with propensity score matched analysis and visualized oocyte and embryo manifestation, and further showing impaired CLBR and implantation condition of pSS patients, so we have more reason to believe that the pSS patients did suffer from deteriorated pregnancy outcomes.

Furthermore, the impact of pSS on fertility outcomes may not be limited to pregnancy outcomes. Women with pSS could experience amenorrhea or menstrual disorders significantly more often than normal women [19]. And not only that, but studies reported that pSS mothers may experience a delayed pregnancy, which means giving birth at an older age than normal ones [9], this in turn leads to a decline in ovarian reserve, and further make it increasingly difficult for them to conceive. Hence, we still emphasize the need for a combination of pSS treatments, a comprehensive fertility evaluation, and ART treatments for a comprehensive range of therapeutic options. Additionally, many prior studies reported that pSS may be associated with preterm birth, low birthweight and neonatal defects especially neonatal heart block [34, 36, 37]. A substantial correlation between congenital heart block and anti-Ro/SS-A and anti-La/SS-B have been demonstrated in previous studies [38–40]. A meta-analysis of nine studies suggested that SS was highly associated with preterm birth (RR = 2.27, 95% CI 1.46‒3.52) and low birth mass (RR = 1.99, 95% CI 1.34‒2.97) [37], and this conclusions were also similar to our results. Additionally, a literature review including seven studies by Sara et al. showed that pSS patients could accompanied with distinct increases of spontaneous abortions, preterm deliveries and cesarean section [12], and presented that pSS was responsible for adverse clinical outcomes. It's worth noting that a newborn in our pSS group performed one degree of conduction block and the maternal SSA antibody titer was as high as 1:1000 during pregnancy, which gives us more alert in providing pregnancy instruction in pSS patients with high SSA antibody titer. Although the other neonatal outcomes were essentially similar to the normal group, due to sample size limitations, more large-scale evidence and follow-up also need to be conducted in future studies.

### Strength and limitation

#### Strength:

The present study first provided a new perspective on the impact of pSS on female fertility in terms of oocyte and embryo viability, while prior researchers had usually concentrated on obstetric and fetal complications of pSS. Meanwhile, the study also took the ovarian reserve and clinical pregnancy outcomes of pSS patients into consideration, which was a comprehensive analysis of the impact of pSS on female fertility. In addition, this study combined data from several hospitals, and the corresponding comparison group excluded possible interferences such as a history of endometriosis, PCOS, and ovarian surgery by PSM matching. Lastly, both conservative and optimistic CLBRs were calculated to exhibit the ART outcomes that patients were most concerned about.

#### Limitation:

This study also has several certain limitations. Firstly, it’s a retrospective study, which is always relevant to an inevitable risk of bias. Secondly, two patients had been treated with methotrexate therapy, which may also potentially affect their ovarian function. Lastly, due to its special population selection, the sample size of this study is not quite large.

## Conclusion

In conclusion, this study showed that women with a clinical diagnosis of pSS presented deteriorated oocyte and embryonic development, even in remission. Also, women with pSS exhibited diminished ovarian reserve and impaired clinical pregnancy outcomes. This study offers evidence to support future research on the effects of pSS and other autoimmune diseases on female fertility, and it also calls for more attention to be given to these patients by reproductive physicians and rheumatologists, as well as timely intervention for fertility impairment. Comprehensive fertility assessment and followed individualized fertility guidance are recommended for patients with a history of pSS. More large-scale and multi-center studies are required to further investigate the mechanisms of the impact of primary Sjögren syndrome on female fertility.

## Data Availability

The data underlying this article are available in the article and its supplementary material. Further inquiries can be directed to the corresponding authors.

## References

[1] Deroux A, Dumestre-Perard C, Dunand-Faure C, Bouillet L, Hoffmann P (2017). Female Infertility and Serum Auto-antibodies: a Systematic Review. Clin Rev Allergy Immunol.

[2] Gurka G, Rocklin RE (1987). Reproductive immunology. JAMA.

[3] Brito-Zerón P, Baldini C, Bootsma H, Bowman SJ, Jonsson R, Mariette X (2016). Sjögren syndrome. Nat Rev Dis Primers.

[4] Ramos-Casals M, Brito-Zerón P, Sisó-Almirall A, Bosch X (2012). Primary Sjogren syndrome. BMJ.

[5] Marder W, Littlejohn EA, Somers EC (2016). Pregnancy and autoimmune connective tissue diseases. Best Pract Res Clin Rheumatol.

[6] Spinillo A, Beneventi F, Locatelli E, Ramoni V, Caporali R, Alpini C (2016). Early, Incomplete, or Preclinical Autoimmune Systemic Rheumatic Diseases and Pregnancy Outcome. Arthritis Rheumatol.

[7] Upala S, Yong WC, Sanguankeo A (2016). Association between primary Sjögren's syndrome and pregnancy complications: a systematic review and meta-analysis. Clin Rheumatol.

[8] Manolis AA, Manolis TA, Melita H, Manolis AS (2020). Congenital heart block: Pace earlier (Childhood) than later (Adulthood). Trends Cardiovasc Med.

[9] Hussein SZ, Jacobsson LTH, Lindquist PG, Theander E (2011). Pregnancy and fetal outcome in women with primary Sjogren's syndrome compared with women in the general population: a nested case-control study. Rheumatology (Oxford).

[10] Karakus S, Sahin A, Durmaz Y, Aydin H, Yildiz C, Akkar O (2017). Evaluation of ovarian reserve using anti-müllerian hormone and antral follicle count in Sjögren's syndrome: Preliminary study. J Obstet Gynaecol Res.

[11] Ballester C, Grobost V, Roblot P, Pourrat O, Pierre F, Laurichesse-Delmas H (2017). Pregnancy and primary Sjögren's syndrome: management and outcomes in a multicentre retrospective study of 54 pregnancies. Scand J Rheumatol.

[12] De Carolis S, Salvi S, Botta A, Garofalo S, Garufi C, Ferrazzani S (2014). The impact of primary Sjogren's syndrome on pregnancy outcome: our series and review of the literature. Autoimmun Rev.

[13] Choudhry HS, Hosseini S, Choudhry HS, Fatahzadeh M, Khianey R, Dastjerdi MH (2022). Updates in diagnostics, treatments, and correlations between oral and ocular manifestations of Sjogren's syndrome. Ocul Surf.

[14] Zhu L, Xi Q, Zhang H, Li Y, Ai J, Jin L (2013). Blastocyst culture and cryopreservation to optimize clinical outcomes of warming cycles. Reprod Biomed Online.

[15] Ferraretti AP, La Marca A, Fauser BCJM, Tarlatzis B, Nargund G, Gianaroli L (2011). ESHRE consensus on the definition of 'poor response' to ovarian stimulation for in vitro fertilization: the Bologna criteria. Hum Reprod.

[16] Yang Q, Hu J, Wang M, Li Z, Huang B, Zhu L (2022). Early Cervical Lesions Affecting Ovarian Reserve and Reproductive Outcomes of Females in Assisted Reproductive Cycles. Front Oncol.

[17] Chambers GM, Paul RC, Harris K, Fitzgerald O, Boothroyd CV, Rombauts L (2017). Assisted reproductive technology in Australia and New Zealand: cumulative live birth rates as measures of success. Med J Aust.

[18] Xu B, Chen Y, Geerts D, Yue J, Li Z, Zhu G (2018). Cumulative live birth rates in more than 3,000 patients with poor ovarian response: a 15-year survey of final in vitro fertilization outcome. Fertil Steril.

[19] Haga HJ, Gjesdal CG, Irgens LM, Ostensen M (2005). Reproduction and gynaecological manifestations in women with primary Sjögren's syndrome: a case-control study. Scand J Rheumatol.

[20] Zhu YZ, Zhong JX, Dong LL (2023). Menstrual and Reproductive Characteristics of Patients with Primary Sjogren's Syndrome: A 7-year Single-center Retrospective Study. Curr Med Sci.

[21] Angley M, Spencer JB, Lim SS, Howards PP. Anti-Müllerian hormone in African-American women with systemic lupus erythematosus. Lupus Sci Med. 2020;7. 10.1136/lupus-2020-000439 PMC7607611 33132225

[22] Forges T, Monnier-Barbarino P, Faure GC, Béné MC (2004). Autoimmunity and antigenic targets in ovarian pathology. Hum Reprod Update.

[23] Szabó K, Jámbor I, Szántó A, Horváth IF, Tarr T, Nakken B (2021). The Imbalance of Circulating Follicular T Helper Cell Subsets in Primary Sjögren's Syndrome Associates With Serological Alterations and Abnormal B-Cell Distribution. Front Immunol.

[24] Wei C, Jenks S, Sanz I (2015). Polychromatic flow cytometry in evaluating rheumatic disease patients. Arthritis Res Ther.

[25] Ainola M, Porola P, Takakubo Y, Przybyla B, Kouri VP, Tolvanen TA (2018). Activation of plasmacytoid dendritic cells by apoptotic particles - mechanism for the loss of immunological tolerance in Sjögren's syndrome. Clin Exp Immunol.

[26] Konttinen YT, Stegajev V, Al-Samadi A, Porola P, Hietanen J, Ainola M (2015). Sjögren's syndome and extragonadal sex steroid formation: a clue to a better disease control?. J Steroid Biochem Mol Biol.

[27] McCoy SS, Sampene E, Baer AN (2020). Association of Sjögren's Syndrome With Reduced Lifetime Sex Hormone Exposure: A Case-Control Study. Arthritis Care Res (Hoboken).

[28] Johnson EO, Kostandi M, Moutsopoulos HM (2006). Hypothalamic-pituitary-adrenal axis function in Sjögren's syndrome: mechanisms of neuroendocrine and immune system homeostasis. Ann N Y Acad Sci.

[29] Mavragani CP, Fragoulis GE, Moutsopoulos HM (2012). Endocrine alterations in primary Sjogren's syndrome: an overview. J Autoimmun.

[30] Orbach H, Shoenfeld Y (2007). Hyperprolactinemia and autoimmune diseases. Autoimmun Rev.

[31] Mao R, Wang X, Long R, Wang M, Jin L, Zhu L (2023). A new insight into the impact of systemic lupus erythematosus on oocyte and embryo development as well as female fertility. Front Immunol.

[32] Priori R, Gattamelata A, Modesti M, Colafrancesco S, Frisenda S, Minniti A (2013). Outcome of Pregnancy in Italian Patients with Primary Sjögren Syndrome. J Rheumatol.

[33] Skopouli FN, Papanikolaou S, Malamou-Mitsi V, Papanikolaou N, Moutsopoulos HM (1994). Obstetric and gynaecological profile in patients with primary Sjogren's syndrome. Ann Rheum Dis.

[34] Haga H-J, Gjesdal CG, Koksvik HS, Skomsvoll JF, Irgens LM, Östensen M. Pregnancy Outcome in Patients with Primary Sjögren’s Syndrome. A Case-Control Study. J Rheumatol. 16142869

[35] Lao M, Luo G, Dai P, Zhang X, Peng M, Chen Y et al*.* Pregnancy Outcomes in Patients with Primary Sjögren’s Syndrome Undergoing Assisted Reproductive Therapy: A Multi-center Retrospective Study. Rheumatol Ther. 2023. 10.1007/s40744-023-00608-3 PMC10654308 37875747

[36] Barros T, Braga J, Abreu M, Brandão M, Farinha F, Marinho A (2022). Sjögren’s syndrome and pregnancy: a Portuguese case-control study. Reumatologia.

[37] Geng B, Zhang K, Huang X, Chen Y (2022). A meta-analysis of the effect of Sjögren′s syndrome on adverse pregnancy outcomes. Clinics.

[38] Gleicher N, Elkayam U (2013). Preventing congenital neonatal heart block in offspring of mothers with anti-SSA/Ro and SSB/La antibodies: a review of published literature and registered clinical trials. Autoimmun Rev.

[39] Martínez-Sánchez N, Pérez-Pinto S, Robles-Marhuenda Á, Arnalich-Fernández F, Martín Cameán M, Hueso Zalvide E (2017). Obstetric and perinatal outcome in anti-Ro/SSA-positive pregnant women: a prospective cohort study. Immunol Res.

[40] Zuppa AA, Riccardi R, Frezza S, Gallini F, Luciano RMP, Alighieri G (2017). Neonatal lupus: Follow-up in infants with anti-SSA/Ro antibodies and review of the literature. Autoimmun Rev.

